# A functional linear modeling approach to sleep–wake cycles in dogs

**DOI:** 10.1038/s41598-020-79274-2

**Published:** 2020-12-17

**Authors:** Hope J. Woods, Ming Fei Li, Ujas A. Patel, B. Duncan X. Lascelles, David R. Samson, Margaret E. Gruen

**Affiliations:** 1 Department of Clinical Sciences, College of Veterinary Medicine, North Carolina State University, Raleigh, NC USA; 2 Department of Anthropology, University of Toronto, Toronto, ON Canada; 3 Department of Anthropology, University of Toronto Mississauga, Mississauga, ON Canada; 4 Translational Research in Pain (TRiP) Program, Department of Clinical Sciences, College of Veterinary Medicine, North Carolina State University, Raleigh, NC USA; 5 Comparative Pain Research and Education Centre, College of Veterinary Medicine, North Carolina State University, Raleigh, NC USA; 6 Comparative Medicine Institute, Department of Clinical Sciences, College of Veterinary Medicine, North Carolina State University, 1060 William Moore Drive, Raleigh, NC 27612 USA; 7 Thurston Arthritis Centre, UNC School of Medicine, Chapel Hill, NC USA; 8 Department of Anaesthesiology, Center for Translational Pain Research, Duke University, Durham, NC USA

**Keywords:** Biological techniques, Animal behaviour, Animal physiology, Dementia, Sleep disorders

## Abstract

The study of companion (pet) dogs is an area of great translational potential, as they share a risk for many conditions that afflict humans. Among these are conditions that affect sleep, including chronic pain and cognitive dysfunction. Significant advancements have occurred in the ability to study sleep in dogs, including development of non-invasive polysomnography; however, basic understanding of dog sleep patterns remains poorly characterized. The purpose of this study was to establish baseline sleep–wake cycle and activity patterns using actigraphy and functional linear modeling (FLM), for healthy, adult companion dogs. Forty-two dogs were enrolled and wore activity monitors for 14 days. FLM demonstrated a bimodal pattern of activity with significant effects of sex, body mass, and age; the effect of age was particularly evident during the times of peak activity. This study demonstrated that FLM can be used to describe normal sleep–wake cycles of healthy adult dogs and the effects of physiologic traits on these patterns of activity. This foundation makes it possible to characterize deviations from normal patterns, including those associated with chronic pain and cognitive dysfunction syndrome. This can improve detection of these conditions in dogs, benefitting them and their potential as models for human disease.

## Introduction

Companion dogs offer a translational opportunity to study sleep, and gain a greater understanding of the conditions and disorders contributing to sleep disturbance in humans, such as those arising from chronic pain, ageing, and cognitive dysfunction. As a result of domestication, companion dogs are members of the household, sharing an environment, routine, and, to an extent, diet with their humans^1–3^. Companion dogs, as a previously established, housed, and cared for population, also offer an ethical opportunity for comparative research^1^. Sleep disturbance can be assessed by measuring activity, but in order to do this effectively, activity variation over the day—and the factors that affect it—need to be fully understood.

Some data are available on overall activity and factors affecting activity in companion dogs. Activity studies reveal variation in routines for dogs in the home that track with differing human schedules during timeframes such as weekdays and weekends^3–8^. In this manner, companion dogs offer a unique comparative model as activity fluctuates with human-based changes to routine. This human influence on activity patterns is evident in studies of laboratory-housed, shelter, and companion animals; companion dogs are more active compared to laboratory-housed dogs^7^ and achieve more rest compared to shelter dogs^9, 10^, due to greater human interaction. It is evident that environmental context also influences activity^11^ and is an important consideration when studying activity in a comparative context^5,7,8,11^. Some work has been performed looking at individual dog factors influencing activity levels in dogs. Age has been shown to decrease total activity^12^, and to decrease the response (in terms of increased activity) to analgesics administered to dogs suffering pain^13^. Body weight has been shown to have some effects on total activity^14^. However, there is no information on how these factors affect activity profiles, or patterns, over the course of a 24-h period.

Companion dogs offer a distinctive perspective when studying sleep patterns, not only due to shared environmental influences in the home, but also similarities shared by humans and dogs in sleep architecture and sleep–wake cycles^4,15–20^. Both species cycle through phases of wakefulness, drowsiness, rapid eye movement (REM) sleep, and non-rapid eye movement (NREM) sleep^4,15,16,20,21^. As with activity, companion dogs and humans follow a similar circadian rhythm and diurnal sleep pattern^8,21,22^. However, the majority of our understanding of dog sleep patterns comes from studies conducted only at night, or are based on older observational studies of dogs whose primary housing is no longer comparable to the majority of pet dogs^23^. Indeed, the most frequently cited study of dog sleep (conducted by Adams & Johnson in 1993)^23^ is based on observation of twenty-four dogs, eighteen of whom were videotaped during the night (not during the day). All of the dogs videotaped slept outdoors or were housed in a laboratory; none of them slept with humans. This work, while critically important, no longer reflects the manner in which most pet dogs are now housed, as they are kept primarily inside homes, and often in their owners’ bedroom or bed^23^. As technology and monitoring equipment have evolved, a re-evaluation of sleep patterns in dogs is needed.

Further, companion dogs have been put forward as valuable translational models as understanding naturally-occurring disease in dogs can benefit both dogs and humans^2,24–29^. As a translational model of sleep disturbance in humans, dogs allow researchers to study the natural and complex contributing factors of disease, unlike in experimentally induced or transgenic rodent models where aspects of a disease are artificially induced^2,26^. Diseases associated with ageing and cognitive dysfunction of humans naturally occur in dogs across the lifespan in a similar manner^27^; the progression of age-related cognitive decline and comorbidities offers study of environmental, dietary, and longitudinal effects, unlike induced rodent models where changes occur over a brief time course^2,3,28,30^. Researchers have noted sleep disturbances as a result of ageing, cognitive dysfunction, and chronic pain for humans and dogs^31–37^. Takeuchi and Harada^37^ concluded that sleep pattern changes, such as decreased nighttime sleep efficiency, are a result of ageing in laboratory-housed dogs, comparable to humans. It has also been established that sleep and pain hold a bidirectional relationship, in which sleep disturbance may be a result of sleep deficiency enhancing pain or the presence of pain disturbing sleep^31–33,38^. Knazovicky et al.^34^ examined nighttime activity of companion dogs with osteoarthritis under blinded treatment and placebo conditions; using an owner questionnaire, they found that chronic pain impacts quality of sleep contributing to sleep disturbance. These subjective findings were subsequently supported by actigraphy^38^. With these few latter exceptions, the findings regarding age- or disease-associated changes in daily sleep patterns in dogs have all been conducted on laboratory-housed dogs. To accurately examine the effects of ageing, cognitive dysfunction, and chronic pain on the sleep–wake cycle for companion dogs as a human translation model, a comparative baseline measure must first be established.

Actigraphy is a valid and reliable method commonly used to assess rest in human sleep studies and has also been shown to provide an accurate measurement of sleep–wake cycles in dogs^22,39,40^. John et al.^41^ used polygraphy and actigraphy in narcoleptic dogs to demonstrate that actigraphy was able to determine periods of sleep and wake. Actigraphy (through collar-mounted activity monitors) provides a measurement of activity patterns^22,39,42^ and requires no prior training or familiarization for both dogs and their humans. The output from these monitors show rest and activity patterns and may be used to estimate behavioral sleep as previously described^22^. The purpose of the present study is to establish baseline sleep–wake cycle and activity patterns with the use of actigraphy and a novel analytic method, functional linear modeling, for healthy, adult companion dogs who live in the home. We hypothesize that this method will support previously described diurnal patterns and allow for more detailed exploration of the variables affecting behavioral sleep patterns across the 24-h day than traditional analysis methods.

## Methods

All procedures were conducted with the approval of the North Carolina State University Institutional Animal Care and Use Committee (Protocol #18-053-O) and were in accordance with relevant guidelines and regulations. Dogs were recruited from the area surrounding the North Carolina State University College of Veterinary Medicine (NCSU CVM) via email and social media. Interested owners were given a link to a survey to complete with details about their dog. Eligible dogs had to be between two and nine years of age; this range was chosen to be after social maturity across breeds and prior to the age where significant comorbidities were likely. Dogs could not be receiving any psychoactive medications that could affect sleep, and were required to be generally healthy based on owner report and review of medical records. Preventative medications for flea/tick and heartworm were allowed. Owners were eligible if they were over 18 years old, agreed to complete all paperwork and maintain their normal schedule during the 14 days of the study.

Eligible dogs were brought to the NCSU CVM to be fitted with an activity monitor (accelerometer) mounted to their collar (Actical monitor; Philips Respironics). Owners were given an opportunity to ask questions about the study and provided written informed consent for participation. They then completed a questionnaire designed to assess their dog’s sleep over the last 7 days (Sleep and Nighttime Restlessness Evaluation [SNoRE])^34^. This questionnaire asks owners to rate their dog’s overnight movement, twitching, dreaming, shifting position, vocalizing, and pacing on a scale from 0 = Never to 10 = Constant. Owners were instructed to keep their dog’s activity monitor on at all times over the next 14 days and keep a sleep log for their dog, noting any significant disruptions to their dog’s routine or sleep (or if the collar was found off at any time). As part of a secondary study, owners also indicated the time they delineated as ‘nighttime’ (i.e. the time they went to bed). After 14 days, owners returned to the NCSU CVM to complete a final SNoRE questionnaire, and turn in their paperwork and activity monitors.

Activity monitors were set to collect data in 1-min epochs across the study period. Data from the activity monitors were downloaded using a dedicated reader and software system (ActiLife) prior to analysis. For each day, 1440 data points were included. For functional linear modeling, data were included over the 24-h period. Classification of nighttime and daytime for each dog was needed for the linear mixed effects model; this was accomplished using the times owners indicated that they went to bed each night in their dairy entries.

### Statistical analysis

FLM: Functional linear modeling (FLM) is specifically designed for actigraphy time-series data analysis because it models the response variable as function of time^43^. This analytic technique was used here to characterize and illustrate 24-h sleep–wake patterns of the adult dogs. We used the R package “actigraphy”^44^ to convert the raw activity counts per 1-min epoch into a smoothed functional form (i.e., smoothed curve) using a Fourier expansion. This allows for visualization of temporal differences between dogs based on either categorical variables (e.g., sex) and/or continuous variables (e.g., age, body mass)^43^. First, the observed F statistic is calculated, which compares the activity function for paired individuals (i.e., based on categorical or continuous variables) at each time point. Then, to test for the significance of these differences, we applied a non-parametric permutation test method as it does not rely on distributional assumptions. Specifically, the test compares the *observed* pairing to the calculated proportion of and *permutated* F values and thus significance was calculated by counting the proportion of permutation *F* values that are larger than the *F* statistics for the observed pairing. Here, we used the point-wise test (with 500 permutations) which provides a curve that represents the proportion of permutation *F* values that are larger than the *F* statistic for the observed pairing at each point in the time series^43^. For a detailed explanation on the FLM method, see Wang et al.^43^.

### Data analysis

To assess the drivers of activity in dogs, we built two linear mixed effects models (LMMs) using the following formula:

$${\varvec{M}}{\varvec{o}}{\varvec{d}}{\varvec{e}}{\varvec{l}}\boldsymbol{ }\;0:\boldsymbol{ }daytime \;activity \sim day \;type+age+sex+ body \;mass+|1|ID|$$

$${\varvec{M}}{\varvec{o}}{\varvec{d}}{\varvec{e}}{\varvec{l}}\boldsymbol{ }\;1:\boldsymbol{ }nightime \;activity \sim day \;type+age+sex+ body \;mass+|1|ID|$$

The formula interogates whether the response variable *activity* (Model 0 = daytime activity, Model 1 = nighttime activity) is a function of *day type* (weekday versus weekend*)*, *age, sex* (male versus female), and *body mass*. To control for repeated measures of individuals we included “ID” as a random effect (see above formula). Both response variables were positively skewed, thus we used log-transformed values to meet model assumptions. We first used the model selection tool *dredge* to generate candidate models, then we averaged models with ∆AIC < 10 using the *MuMIn* package^45^ and we report the conditional coefficients, as it pools less certain estimates from more certain estimates^46^. The model was built and analyzed using the *lme4* package^47^ in R Version 4.0.1 (R Core Team, R: A language and environment for statistical computing. R Foundation for Statistical Computing, 2020). Statistical inferences were made using standardized coefficient estimates with shrinkage and 95% confidence intervals.

## Results

Eighty-four people completed the screening questionnaire; responses were reviewed in an attempt to balance for sex, age, and size range for the dogs. Forty-three dogs were enrolled into the study; 22 females and 21 males, however one dog was lost to follow-up and no data were obtained; this left 21 females and 21 males completing the study. These 42 dogs had an average age of 5.5 ± 2.3 years (range 2–9 years) and average weight of 23.4 ± 12.2 kg (range 2.7–45.4). At baseline, dogs had a mean SNoRE scale score of 19.4 ± 6.6 ((range 0–34) possible range of 0–60; Supplementary Table 1).

**Table 1:** Summary of conditional model-averaged coefficients from LMM evaluating the effects of day type, age, sex, and body mass on daytime and nighttime activity.

Predictor variable	Estimate (SE)	z-value	*p* value	95% CI
**Daytime model**				
Day type (weekend)	**0.250 (0.028)**	**9.080**	** < 0.001**	**0.196**, **0.304**
Age	− **0.297 (0.099)**	**2.979**	**0.003**	− **0.492**, − **0.102**
Sex (male)	− **0.233 (0.102)**	**2.276**	**0.023**	− **0.435**, − **0.032**
Body mass	− 0.120 (0.111)	1.082	0.279	− 0.338, 0.098
**Nighttime model**				
Day type (weekend)	0.007 (0.034)	0.196	0.845	− 0.061, 0.074
Age	0.027 (0.096)	0.282	0.778	− 0.161, 0.215
Sex (male)	0.035 (0.100)	0.349	0.727	− 0.161, 0.232
Body mass	− 0.096 (0.098)	0.974	0.330	− 0.288, 0.097

Significant effects (*p* < 0.05) are bolded.

Owners of all dogs worked a regular daytime schedule with the majority (35) working five days of the week (of those who did not work five days, three worked four days and three worked seven days of the week). Most owners reported going to bed between 10 and 11 pm on both workdays and non-work days, though more owners reported staying up later in this window on non-work days. The majority of owners reported waking up between 6 and 8am on workdays, and between 7 and 9am on non-work days.

Where dogs slept varied, with 24 (57%) of owners reporting that their dog spent some or all of the night in their bed, while an additional 13 reported that their dog spent some or all of the night in their bedroom. Where dogs were kept during the day also varied. During the day, if no one was home, 8 dogs stayed in a kennel, 11 dogs were semi-restricted (1–3 rooms or sections of the home), 2 dogs accompanied their owner to work, and 21 dogs were free in the home. Twenty-one dogs were the only dog in the home, while 17 dogs had one other dog, 3 had 2 other dogs, and 1 had 3 other dogs in the home.

### Linear mixed effects model (LMM)

For daytime activity, age, sex, and type of day (weekday versus weekend) were significant predictors of activity; during the day, younger dogs were more active than older dogs (*p* = 0.003), female dogs were more active than male dogs (*p* = 0.023), and dogs were more active on weekends than weekdays (*p* < 0.001) (Table 1). No variables were significantly associated with nighttime activity (Table 1).

**Figure 1:**
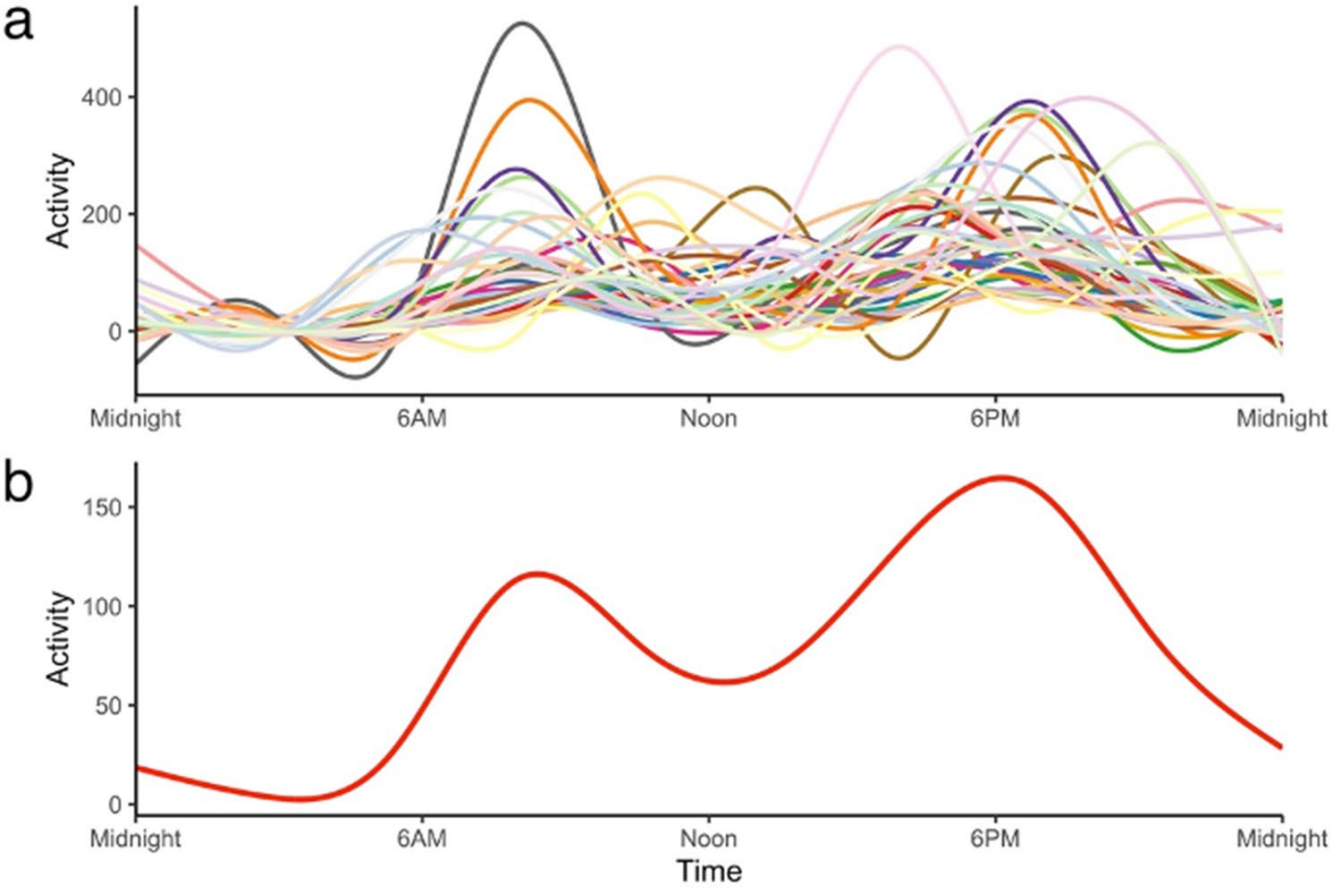
Smoothed line graphs displaying the average activity for (**a**) each dog and (**b**) all dogs.

Figure 1a illustrates the variability of activity patterns between dogs. While dogs differed in their individual patterns of activity, the overall average followed a bimodal diurnal pattern, with peaks at approximately 7am and 7 pm (Fig. 1b). The nadir occurred around noon, but average activity during this time was not as low as during the period between 11 pm and 6am.

### Functional Linear Modeling (FLM)

**Figure 2:**
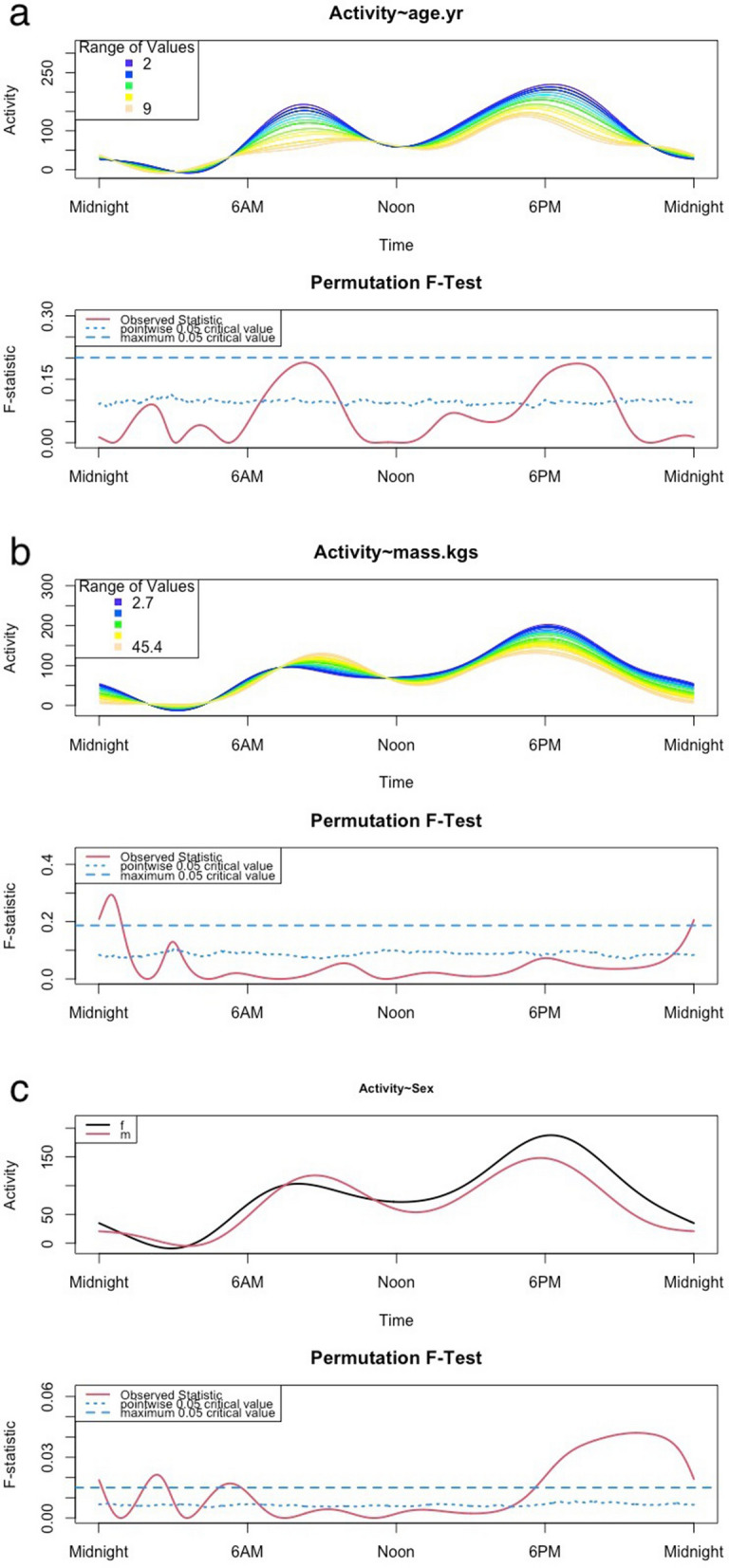
Functional linear models for the 24-h activity pattern showing comparison for (**a**) age, (**b**) mass, and (**c**) sex. Age and mass are continuous variables and sex is categorical. In the lower panel, the dotted blue line represents the point-wise critical value (proportion of all permutation *F* values at a significance level of 0.05) and the solid red line represents the observed *F*-statistic. When the solid line is above the dotted line, it means the groups differ significantly at those time points.

FLM demonstrated that the effect of age on activity was particularly evident during the time of the peaks of activity – younger dogs were significantly more active than older dogs during the period from ~ 7am to 10am and ~ 5:30 pm to 9 pm. Sex and mass both had significant effects in the FLM, with lighter dogs being significantly more active for a short period just after midnight, and females being more active than males during the period from 6 pm to midnight and for brief periods around 3am and 5:30am. (Fig. 2a–c).

## Discussion

This study used functional linear modeling to describe and evaluate the sleep–wake patterns in healthy adult dogs across the 24-h day. Even within this group of healthy dogs, we found that age significantly affected the activity pattern across the day, with effects also seen for mass and sex.

Recent work has provided insights into sleep architecture in dogs^19,21^, with the development of sophisticated non-invasive polysomnography tools. Surprisingly however, this basic question about sleep–wake cycle in dogs had not been fully characterized. Previous studies have focused on laboratory-housed dogs or only nighttime activity, with many studies being decades old and based on small sample sizes. This is particularly vital as dogs have become an increasingly interesting and important model for diseases that affect both dogs and humans. In many of these conditions, including chronic pain and cognitive dysfunction, disrupted or altered sleep has emerged as a key feature. The sleep cycle is integrally related to the daytime activity patterns, and thus the whole 24-h period needs to be evaluated to fully understand normal sleep behavior, and how this is impacted by diseases and conditions, such as cognitive dysfunction.

In this study, we applied two approaches to evaluating the sleep–wake cycle in dogs, and the variables that affect daytime and nighttime activity: a linear mixed effects model, and a functional linear model. In the linear mixed effects model, we found that younger dogs were significantly more active during the day than older dogs, even in this population of healthy adult dogs. Females were more active than males, though this effect was relatively small, compared to the effect of age. In agreement with previous work, there was a significant difference in daytime activity on weekends versus weekdays, with weekend activity being higher. This is not surprising given the influence of human activity on dog activity^5,12^, and highlights the importance of studying activity patterns in pet, rather than laboratory-housed, dogs when establishing this pattern for comparative studies. The linear mixed effects model did not show any significant effects of age, sex, mass, or weekend/weekday on nighttime activity. This may have been affected by our use of the owner-determined start to “nighttime” and an alternative approach could have been to use a standard nighttime threshold.

The use of the owner-determined start to “nighttime” would not affect the findings from the functional linear modeling. As an analytic tool, functional linear modeling has many advantages over previous types of analysis for accelerometer data. This technique incorporates activity counts as they were collected (i.e. original frequency) and allows for more granular evaluations of variable effects across the day. Similar to the linear mixed effects model, the FLM analysis also demonstrated that age was a significant driver in daytime activity, but allowed us to determine when during the day these differences occur, with the most significant differences seen during the periods of peak activity. During these peaks, the younger dogs were significantly more active than the older dogs. Inspection of the curves also suggests a non-significant shift toward an earlier decline in activity and earlier rise time for older dogs. This should be evaluated further, as early rising has been a characteristic of aging in humans^48^. Previous work has shown lower activity in older laboratory-housed dogs. In their study of the effects of age and feeding schedule on diurnal activity patterns in dogs, Zanghi et al.^22^ found that senior dogs (aged 9–11 years) had lower activity than early (aged 1.5–4.5 years) and late adult (aged 7–9 years) dogs across the 24-h day. However, their study also showed a single peak of activity occurring between 9am and 3 pm, the opposite of what we found here^22^; this again demonstrates the need for evaluating these patterns in pet dogs. The differences found for mass and sex are less easily interpretable. There were significant differences during brief periods after midnight where lighter dogs were more active than heavier dogs; further work should be done to explore this finding. Significantly higher activity for female dogs, compared to male dogs, was seen during the evening peak and shorter periods overnight. This has not been previously reported, as many previous studies did not specify the sex of the dogs included^37^ or did not evaluate for sex differences^7,8,22^.

With a solid understanding of the sleep–wake cycle of companion dogs, this ‘comparative model’ can now be further developed to understand the impact on sleep patterns of diseases and conditions, such as cognitive dysfunction. Indeed, one of the cardinal signs of cognitive dysfunction syndrome in dogs is alteration in the sleep–wake cycle^49^. This is despite the fact that what is ‘normal’ is not well-defined, and changes that occur with age, in the absence of cognitive dysfunction, are also poorly characterized in pet dogs. When ‘alterations in sleep–wake cycle’ are noted in dogs with cognitive dysfunction syndrome, it typically refers to daytime sleep with nighttime wakefulness and difficulty sleeping^35,49,50^, however these are likely to be signs of relatively advanced disease. The ability to diagnose cognitive dysfunction in dogs is relatively poor; currently, a large gap exists between the age when dogs in laboratory conditions begin to show deficits in cognitive testing and the age at which pet dogs are typically diagnosed with the condition. The average age when the majority of pet dogs are diagnosed with cognitive dysfunction is 15–16 years^51^, despite evidence of cognitive impairment in laboratory tests of dogs over 6 years of age^52^. Given the importance of sleep–wake cycles in the diagnosis of cognitive dysfunction syndrome, it is possible that early detection of alterations in the sleep–wake cycle could lead to earlier diagnosis of this condition in dogs, both improving their welfare and increasing their relevance as a model for humans with mild cognitive impairment.

The dogs included in this study were considered physically healthy, but were not formally evaluated for conditions that can disrupt sleep, such as chronic pain^34,38^. The SNoRE questionnaire results suggested very little, if any, sleep disturbance, and owners did not report any recent changes in their dogs’ sleep; however, this should be considered a limitation of the study. In addition, this study did not control for where the dogs slept or how many other pets were in the home. Finally, while activity counts have been shown to correlate with sleep periods, quiet rest would register the same way on an accelerometer; we cannot conclude that the absence of activity was always sleep. Accelerometry has been used successfully to evaluate sleep–wake cycles in humans, and actigraphy has been validated in dogs for the demonstration of sleep and wake periods^41^. Additional analytic techniques are being developed to establish rest/sleep versus alert but sedentary behaviors and positions in dogs^39^. Despite the inability to conclusively demonstrate that dogs were sleeping during these periods of inactivity, our results demonstrate the daily pattern of activity in dogs.

Among the advantages of our set-up, FLM allows us to move past the inherent limitations from summary analytic techniques which lack the time-bound detail FLM provides, or from subjective surveys regarding dog sleep. Much remains unknown about dog sleep that could be addressed using this method. For example, little is known about the development of adult-like sleep–wake patterns in dogs. A recent study by Kinsman et al.^53^ has described sleep in puppies over the period between 16 weeks and 12 months; this is an important first look at sleep during this ontogenetic period, however its main limitation was that it depended on surveys of owners. Using survey responses, the study found that daytime sleep decreased over this period; however, features of the dogs’ sleep were missed due to lack of observation. A complementary analysis using accelerometry and FLM would add to our knowledge about the development of adult-like sleep patterns in dogs. Similarly, a comparison of senior and geriatric dogs, with and without cognitive dysfunction, would provide further characterization of alterations in sleep–wake cycles that are associated with this condition. Such work would incrementally build the applicability of companion dogs as a translational model to gain a greater understanding of the conditions or disorders contributing to sleep disturbance in humans.

In conclusion, this study demonstrated that by using FLM, we can determine the normal sleep–wake cycles of healthy adult dogs, and evaluate the effects of age, sex, and body mass on these patterns of activity. With this foundation, we can perform complementary work to better characterize deviations from the normal pattern, including those associated with cognitive dysfunction syndrome. This has the potential to improve detection of cognitive dysfunction syndrome in dogs, benefitting them and their potential as a model for understanding human disease.

## Supplementary information

Supplementary Table 1.

## Data Availability

Data are available from the authors upon reasonable request.
