# Supplementary material for: A functional linear modeling approach to sleep–wake cycles in dogs
Source: Sci Rep. 2020 Dec 17;10:22233. doi: 10.1038/s41598-020-79274-2 (PMC7747556; doi:10.1038/s41598-020-79274-2)
Supplement: Supplementary file 1 — Supplementary Table 1. [file 41598_2020_79274_MOESM1_ESM.pdf]

A functional linear modeling approach to sleep-wake cycles in dogs

Hope J. Woods, Ming Fei Li, Ujas A. Patel, B. Duncan X. Lascelles, David R. Samson, Margaret E. Gruen

Supplementary Table 1. Demographics for study dogs including name, breed, age, sex, and body mass. Average daytime and nighttime per-minute activity counts are shown.

Case	Dog Name	Breed	Age (years)	Sex	Body mass (kg)	Daytime average	Nighttime average
01	Bear	Mix	4	m	36	46.67	8.66
02	Obi	Mix	4	m	6.8	144.75	7.36
03	Beignet	Mix	8	f	25	65.33	10.26
04	Bentley	Cavalier King Charles spaniel	7	m	9.1	152.85	12.52
05	Scout	Maltese	3	f	5.4	205.85	12.05
06	Olive	French bulldog	2	f	4.5	145.74	17.20
07	Memphis	Mix	3	f	23	97.86	8.21
08	Mya	Mix	9	f	11	172.81	19.93
09	Fang	Mix	5	m	30	157.30	41.92
10	Izzy	Jack Russel Terrier	6	f	5.4	161.57	15.46
11	Ethyl	Mix	7	f	14	144.74	6.58
12	Cappie	Hound	8.5	m	23	44.79	16.22
13	Violet	Mix	7	f	32	139.10	13.21
14	Maddie	Mix	8.5	f	23	112.27	3.34
15	Addie	Mix	5	f	16	67.32	11.76
17	Gibita	Shetland sheepdog	9	f	14	89.81	19.26
18	Daisy	Mix	9	f	14	54.65	12.44
19	Ridge	Rhodesian Ridgeback	4.5	m	43	156.39	10.18
20	Tully	Mix	3	f	18	173.81	21.12
21	Chloe	Mix	9	f	23	71.56	11.38
22	Carl	Rottweiler	5	m	45	67.24	7.36
23	Bruce	Labrador retriever	6	m	45	98.99	17.25
24	Eli	Am. Staff. Terrier	8	m	27	56.00	3.18
25	Batou	German Shepherd Dog	4	m	41	136.70	18.56
26	Rue	Plott hound	2.5	f	27	84.75	2.83
27	Sully	Mix	5	m	6.8	124.40	26.32
28	Schumie	Am. Staff. Terrier	3.5	f	23	166.40	2.81
29	Dizzi	Mix	8	f	16	107.00	7.56
30	Kaipo	Pitbull	3	m	34	92.20	4.87
31	McCoy	Golden retriever	8	m	30	84.95	11.78

32	Lake Sparkle	Mix	7	f	30	82.05	11.83
33	Tanzy	Labrador retriever	3	f	34	127.32	26.88
34	George	Pug	4	m	6.8	46.89	16.11
35	Louis	Mix	6.5	m	34	40.88	8.82
36	Loki	Doberman	2	m	41	172.80	13.49
37	Benny	Mix	2.5	m	27	103.41	8.72
38	Angus	Mix	8	m	34	110.86	23.63
39	Taki	Mix	5	m	27	107.24	20.94
40	Gus Gus	Mix	4	m	32	97.51	3.85
41	Rocket Man	Chihuahua	8	m	2.7	79.38	21.67
42	Ginger	Standard Poodle	2.5	f	32	203.38	21.47
43	Lucy	Mix	4	f	13	195.26	32.44
